# Endogenous β-hydroxybutyrate and the risk of cognitive decline: a nested case-control study in the UK Biobank cohort

**DOI:** 10.3389/fnagi.2026.1768532

**Published:** 2026-02-11

**Authors:** Youngmi Eun, John C. Newman, Ji Hyun Lee, Se-Hong Kim, Ha-Na Kim

**Affiliations:** 1 Department of Family Medicine, Yeouido St. Mary’s Hospital, College of Medicine, The Catholic University of Korea, Seoul, Republic of Korea; 2 Division of Geriatrics, School of Medicine, University of California, San Francisco, San Francisco, CA, United States; 3 Buck Institute for Research on Aging, Novato, CA, United States; 4 Department of Dermatology, Seoul St. Mary’s Hospital, College of Medicine, The Catholic University of Korea, Seoul, Republic of Korea; 5 Department of Family Medicine, St. Vincent’s Hospital, College of Medicine, The Catholic University of Korea, Seoul, Republic of Korea

**Keywords:** cognitive decline, cognitive domains, ketone bodies, UK Biobank, β-hydroxybutyrate

## Abstract

**Introduction:**

β-hydroxybutyrate (BHB) has been linked to improved cognitive function via enhanced cerebral metabolism and anti-inflammatory effects. However, evidence on the relationship between endogenous plasma BHB levels and cognitive decline remains limited. This study investigated whether higher plasma BHB levels are associated with reduced cognitive decline in middle-aged and older adults.

**Methods:**

We conducted a nested case–control study within the UK Biobank prospective cohort. Among 4,653 participants with baseline plasma BHB measurements and cognitive assessments at baseline and follow-up, 2,143 cases of cognitive decline were matched to 2,143 controls by age, sex, and polygenic risk score for Alzheimer’s disease. Plasma BHB concentrations were measured by nuclear magnetic resonance spectroscopy. Cognitive function was assessed across five domains, and a global cognition score was derived through factor analysis. Cognitive decline was defined as a decrease in the global cognition score from baseline to follow-up. Conditional logistic regression and restricted cubic spline models were used to examine the associations between plasma BHB levels and cognitive decline, adjusting for demographic, lifestyle, and clinical factors.

**Results:**

Plasma BHB concentrations were not significantly associated with global cognitive decline after covariate adjustment. However, higher plasma BHB tertiles were associated with a slower decline in fluid intelligence (adjusted OR for tertile 1 vs. 3, 0.83; 95% CI, 0.72–0.97).

**Discussion:**

Endogenous plasma BHB was not significantly associated with global cognitive decline but was inversely related to decline in fluid intelligence. Further studies are needed to clarify BHB’s role in cognitive aging.

## 1 Introduction

The global prevalence of dementia is expected to increase with advancing age (Prince et al., 2013). Dementia results in cognitive impairment and neurobehavioral symptoms that substantially affect daily life (Livingston et al., 2020), and induce a high burden of disability, mortality, and socioeconomic costs related to care (Hurd et al., 2013).

Many factors, including age and genetics, are both risk factors and predictors of dementia and subtle cognitive decline that can occur in the decades before dementia is diagnosed (Dominguez et al., 2021; Livingston et al., 2020; Norton et al., 2014). Ketone bodies are endogenous fuels produced by the liver when glucose availability is low, such as during fasting and starvation, a process called ketogenesis (Newman and Verdin, 2017), and include acetoacetate, β-hydroxybutyrate (BHB), and acetone, of which BHB is the most abundant circulating ketone body (Lincoln et al., 1987). Beyond its energetic function, BHB has been linked to cell signaling, inflammatory modulation, and epigenetic regulation (Newman and Verdin, 2017).

Studies have highlighted the relationship between high BHB levels induced by a ketogenic diet or supplemental BHB and improved cognitive function (Myette-Cote et al., 2022; Reger et al., 2004; Roberts et al., 2017) via increased cerebral metabolism as well as by modulating brain inflammation (Polito et al., 2023) and the regulation of microglial metabolism (Jin et al., 2023); however, little is known about the influence of endogenous BHB status on cognition, without interventions that increase body BHB status.

We hypothesized that higher plasma BHB levels within the physiological range observed in the fed, non-fasting state, without conditions or interventions intended to increase BHB, are associated with a lower risk of cognitive decline, independent of age, sex, and genetic predisposition to Alzheimer’s disease. This study aimed to examine the risk of cognitive decline according to plasma BHB levels in middle-aged and older adults using a nested case–control design based on a prospective UK Biobank study.

## 2 Materials and methods

### 2.1 Data sources

The UK Biobank was approved by the North West Multicentre Research Ethics Committee (11/NW/0382), and informed consent was obtained from all participants. This study used data from the UK Biobank after an application was approved (ID 87759), which has not been deposited in a public repository and is available after the approval of an application at https://www.ukbiobank.ac.uk. The analyses were approved by the Institutional Review Board of the Catholic University of Korea (VC24ZISI0282). This study was conducted in accordance with the Declaration of Helsinki.

### 2.2 Study design and population

The UK Biobank is a population-based prospective cohort study and open resource conducted in the UK across England, Scotland, and Wales, which recruited 502,235 volunteers aged 40 to 69 years at baseline. Baseline measurements were obtained between 2006 and 2010, and a follow-up examination was performed between 2014 and 2020, including physical measurements, biological samples, and responses to touchscreen questionnaires (sociodemographic factors, early life exposures, family history, general health and disabilities, cognitive, and psychological state, and lifestyle risk markers).

Of the 4,731 participants with measured plasma levels of BHB at baseline and available cognitive test data values at baseline and follow-up, those who had Alzheimer’s disease or other types of dementia at baseline (*n* = 7), were missing a polygenic risk score (PRS) for Alzheimer’s disease at baseline (*n* = 50), with type 1 diabetes (*n* = 20), and who were pregnant (*n* = 1) were excluded, leaving eligible 4,653 participants for the nested case–control study.

**Figure 1:**
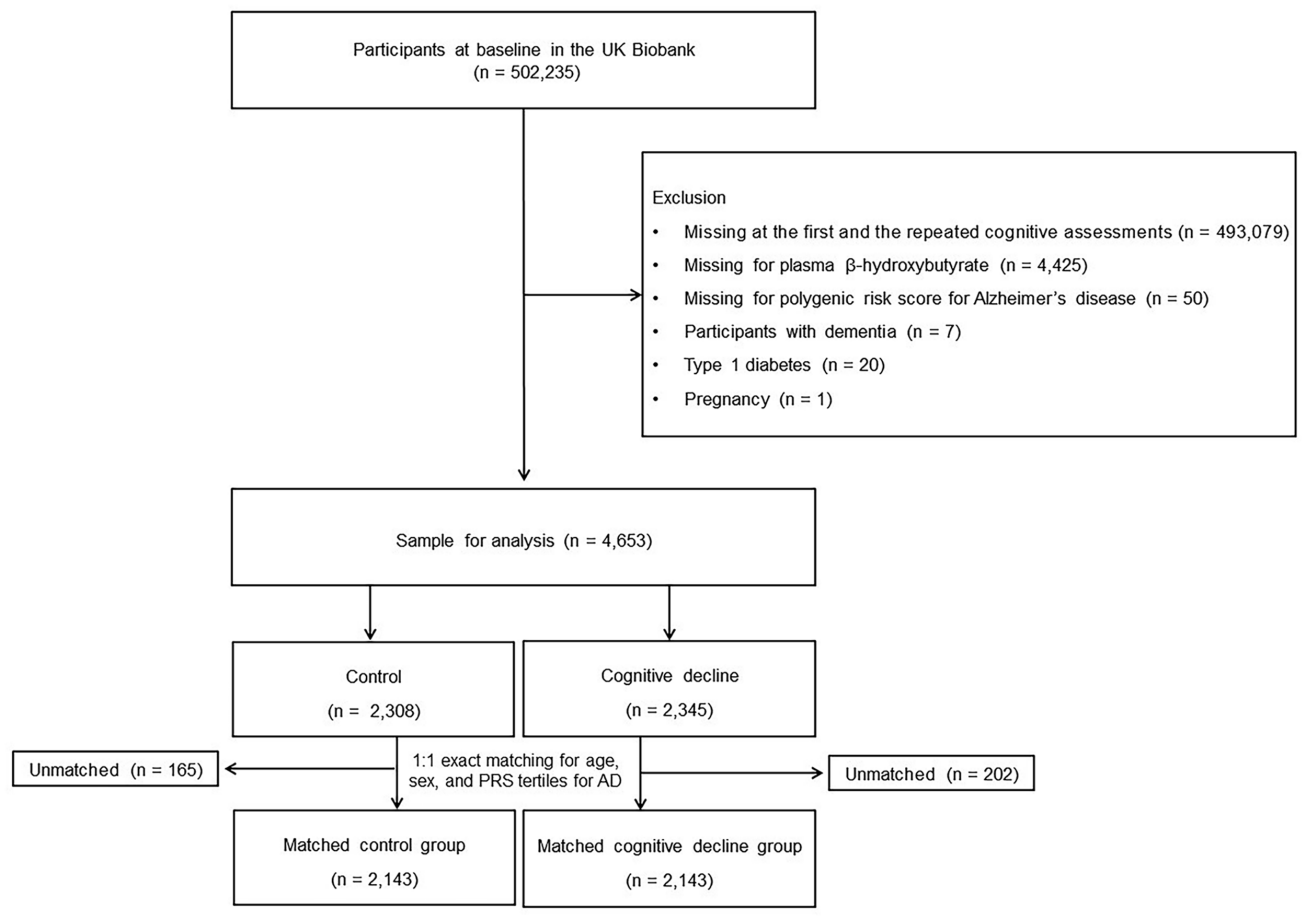
Flowchart of the study population.

Cases in the study cohort were defined as those with cognitive decline during follow-up after cohort entry. The participants with cognitive decline in each case were matched 1:1 with controls for age (±5 years) at the date of cohort entry, sex, and the tertiles of PRS for Alzheimer’s disease. Controls were randomly selected to minimize selection bias. Ultimately, 2,143 matched cases and 2,143 matched controls were included in the analysis (Figure 1).

### 2.3 Exposure

Plasma BHB levels available from UK Biobank data were used at baseline. These values were measured using a high-throughput nuclear magnetic resonance-based metabolic biomarker profiling platform developed by Nightingale Health Ltd. (Helsinki, Finland). Non-fasting EDTA plasma samples were collected at baseline recruitment (2006–2010) and prepared in 96-well plates by the UK Biobank Laboratory (Stockport, United Kingdom) (Elliott et al., 2008). Plasma samples were shipped to Nightingale Health and stored in a freezer at −80 °C. Before testing, the frozen samples were slowly thawed overnight at 4 °C mixed gently, and centrifuged (3 min, 3,400 × *g*, 4 °C) to remove any possible precipitate. Aliquots of each sample were transferred into 3-mm outer-diameter nuclear magnetic resonance tubes and mixed in a 1:1 ratio with phosphate buffer (75 mM Na_2_HPO_4_ in 80%/20% H_2_O/D_2_O, pH 7.4, including 0.08% sodium 3-[trimethylsilyl] propionate-2,2,3,3-d4 and 0.04% sodium azide) automatically with an automated liquid handler (PerkinElmer Janus Automated Workstation). All analyses were performed according to the standard operating procedures of the Nightingale Health EN ISO 13485-certified Quality Management System (Julkunen et al., 2023).

### 2.4 Outcome

At both baseline and the second follow-up, cognitive function assessments were administered via touch-screen questionnaires on a computer. The tests covered five cognitive domains: pairs matching (the number of errors when recalling positions of matching cards), reaction time (mean time to match cards correctly), prospective memory (successfully carrying out an instruction after a filled delay), fluid intelligence (the number of correct answers to logic/reasoning-type questions within a two-minute period), and numeric memory (the maximum numeric string recalled correctly). For numeric memory, prospective memory and fluid intelligence tests, higher raw scores indicate better cognitive function, while for the pairs matching and reaction time tests, higher raw scores indicate worse cognitive function (Fawns-Ritchie and Deary, 2020; Lyall et al., 2016).

Cognitive decline was defined as cases in which the follow-up global cognition score minus the baseline global cognition score resulted in a negative value. The values from all five cognitive domains were used to obtain a measure of global cognition score. The values of the pairs matching and reaction time tests were reversed for ease of comprehension and showed negative skew after reversing, so a square-root transformation was applied. All cognitive variables were converted to Z-scores. *Z*-scores were calculated by subtracting the sample mean and dividing by the standard deviation derived from baseline measurements, with the same parameters applied to follow-up assessments, in order to place all cognitive test scores on a common scale, ensure equal contribution of each domain to factor analysis, and facilitate longitudinal comparability. All cognitive function domains at baseline and follow-up were reduced in dimensionality using factor analysis and averaged to yield baseline and follow-up global cognition scores, respectively, with higher values reflecting better overall cognitive function (Dove et al., 2024; Yang et al., 2024).

### 2.5 Covariates

Information on age, sex, ethnicity, education (college/university degree or higher versus other), household income (60% over or below the median), alcohol consumption frequency (never, currently <3, or ≥3 times/week), smoking status (never, previous, or current), physical activity [sufficient (moderate 150 or more min/week, or vigorous 75 or more min/week) versus other], body mass index [BMI: weight (kilograms) divided by height (meters) squared], and history of comorbidities including hypertension, dyslipidemia, type 2 diabetes mellitus, ischemic heart disease, and cerebrovascular disease was obtained from the baseline visit data and used as covariates. Comorbidities at baseline were determined from medical records and ICD-10 codes used to identify type 2 diabetes (E11), hypertension (I10), dyslipidemia (E78), ischemic heart diseases (I20–25), and cerebrovascular diseases (I60–69). This study used the standard PRS for Alzheimer’s disease constructed by and provided through the UK Biobank showcase (Field ID: 26206), which was calculated for all individuals in the UK Biobank by meta-analyzing multiple external genome-wide association study sources.

### 2.6 Statistical analysis

We used *t*-tests to compare continuous variables and chi-squared tests to compare dichotomous variables. Data are expressed as means (standard deviations) or numbers (percentages). Linear regression analysis was performed with BHB as the independent variable and cognitive function as the dependent variable. The results are presented separately for crude and adjusted models, with adjustments made for age, sex, PRS for Alzheimer’s disease, ethnicity, education, income, smoking, alcohol consumption, physical activity, BMI, type 2 diabetes mellitus, dyslipidemia, hypertension, ischemic heart disease, and cerebrovascular diseases. The mean (standard error) changes in cognitive function values based on the tertiles of BHB are shown in both crude and adjusted models with all of the covariates mentioned above. A conditional logistic regression model was used to evaluate the odds ratios (ORs) of cognitive decline for plasma concentrations of BHB as the exposure accounting for age categories, sex, and PRS tertiles for Alzheimer’s disease as the matching factors and additionally adjusting for ethnicity, education, income, smoking, alcohol, physical activities, BMI, and comorbidities, We performed additional analyses using the unconditional logistic regression model according to the decline in five cognitive domains. Crude and adjusted models were used in these analyses, and 95% confidence intervals (CIs) were calculated. Restricted cubic-spline regression was performed to explore the nonlinear relationship between BHB and cognitive decline, and both the crude and adjusted models were plotted.

SAS ver. 9.4 software (SAS Institute, Cary, NC, USA) and R Studio 2023.12.1 + 402 were used to analyze the data. All statistical analyses were two-sided, and statistical significance was set at *p* < 0.05 was considered statistically significant.

## 3 Results

### 3.1 Characteristics of the study population

From the 4,653 participants with measured plasma levels of BHB at baseline and available cognitive test data values at baseline and follow-up, we matched 2,143 cases [age, 55.38 (7.29) years] with 2,143 controls [age, 55.25 (7.32) years] by 1:1 matching for age (± 5 years), sex, and tertiles of PRS for Alzheimer’s disease. The mean duration of follow-up between the baseline and the second cognitive assessment was 8.2 years (standard deviation, 2.88). Table 1 summarizes the characteristics of the cases and controls. A significant difference in physical activity was observed between individuals with cognitive decline and controls (*p* = 0.012). The mean plasma concentration of BHB in the cognitive decline group did not differ from that in the control group (*p* = 0.808). The scores of global cognition and all cognitive function domains at baseline and follow-up are shown in Supplementary Table 1.

**Table 1:** Characteristics of the study population.

Characteristic	Cognitive decline (*n* = 2,143)	Controls (*n* = 2,143)	*p*
Age, yrs	55.38 (7.29)	55.25 (7.32)	0.540
Age, %			—^*^
40–44	200 (9.3)	200 (9.3)	
45–49	320 (14.9)	320 (14.9)	
50–54	417 (19.5)	417 (19.5)	
55–59	476 (22.2)	476 (22.2)	
60–64	520 (24.3)	520 (24.3)	
≥65	210 (9.8)	210 (9.8)	
Men, %	1,006 (46.9)	1,006 (46.9)	—^*^
Polygenic score for Alzheimer’s disease	0.0165 (0.9898)	0.0034 (0.9936)	0.667
Polygenic score for Alzheimer’s disease, tertiles			—^*^
Tertile 1	−0.9531 (0.3537)	−0.9833 (0.4010)	
Tertile 2	−0.1190 (0.2293)	−0.1139 (0.2259)	
Tertile 3	1.1619 (0.7061)	1.1484 (0.6763)	
Ethnicity, %			0.164
White	2,074 (96.8)	2,078 (97.0)	
Black	10 (0.5)	15 (0.7)	
Asian	34 (1.6)	20 (0.9)	
Other	25 (1.1)	30 (1.4)	
Education, high, %	1,078 (50.3)	1,052 (49.1)	0.729
Low income, 60% below the median, %	208 (9.7)	174 (8.1)	0.187
Alcohol, heavy drinking, %			0.191
Never	112 (5.2)	87 (4.1)	
Current (<3 times/wk)	951 (44.4)	959 (44.8)	
Current (≥3 times/wk)	1,079 (50.4)	1,097 (51.1)	
Current smoking, %	119 (5.6)	121 (5.7)	0.891
Physical activity, sufficient, %	1,098 (51.2)	1,180 (55.1)	0.012
Body mass index, kg/m^2^	26.54 (4.22)	26.48 (4.18)	0.643
Comorbidities, %			
Type 2 diabetes mellitus	91 (4.3)	77 (3.6)	0.271
Dyslipidemia	225 (10.5)	218 (10.2)	0.725
Hypertension	453 (21.1)	450 (21.0)	0.911
Ischemic heart diseases	155 (7.2)	145 (6.8)	0.549
Cerebrovascular diseases	45 (2.1)	35 (1.6)	0.259
Plasma β-hydroxybutyrate levels, mM	0.0598 (0.0570)	0.0602 (0.0613)	0.808
Plasma β-hydroxybutyrate levels, tertiles			0.601
Tertile 1	0.0223 (0.0081)	0.0226 (0.0077)	
Tertile 2	0.0436 (0.0063)	0.0436 (0.0064)	
Tertile 3	0.1151 (0.0712)	0.1126 (0.0809)	

Values are presented as mean (standard deviation) or as number (percentage).

*Matching factors.

*p* values are based on the *t*-test (continuous variables), and chi-squared test (categorical variables).

### 3.2 Dose-response relationships between plasma β-hydroxybutyrate concentrations and cognitive decline across global and domain-specific measures

**Figure 2:**
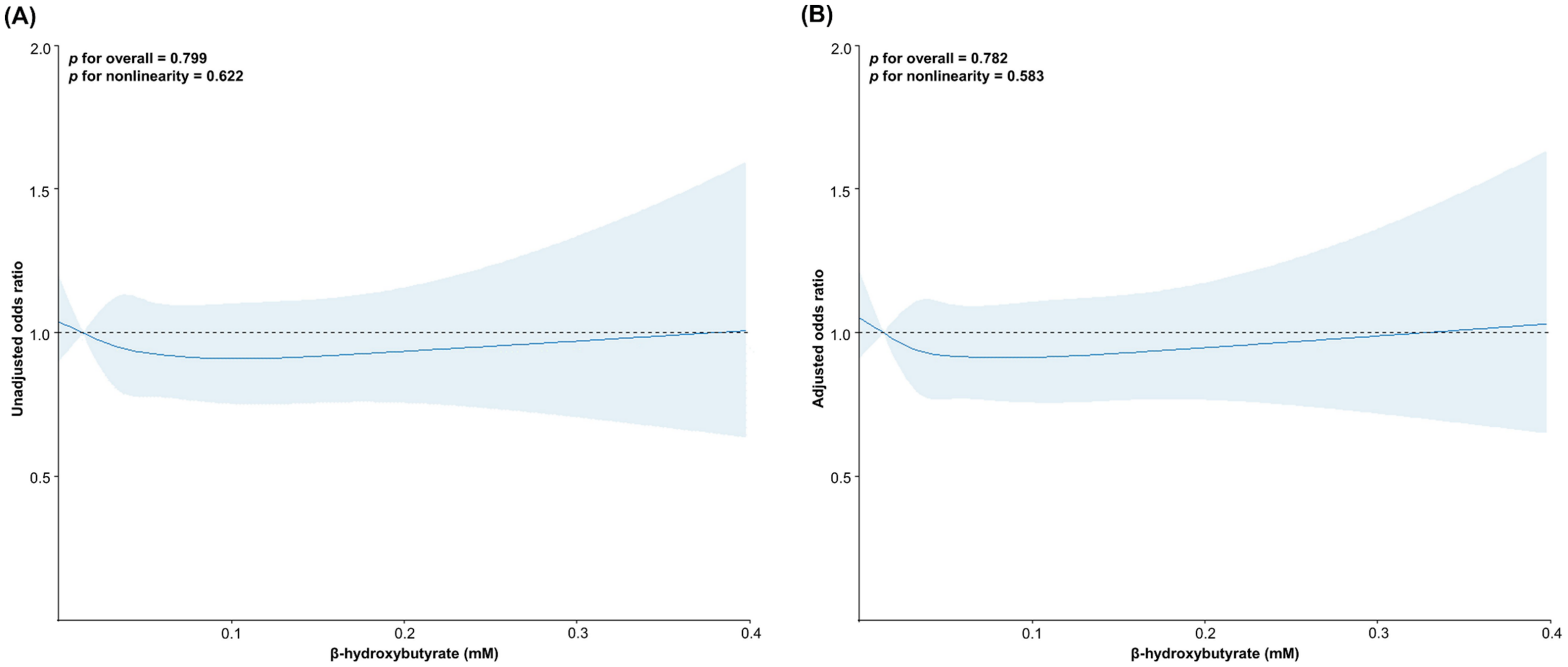
Dose–response analysis of plasma β-hydroxybutyrate concentrations and cognitive decline risk. *p* values were determined using restricted cubic spline regression in unadjusted **(A)** and adjusted models **(B)**. Adjusted for age, sex, polygenic risk score for Alzheimer’s disease, ethnicity, household income, educational level, smoking status, alcohol consumption, physical activity, body mass index, and history of comorbidities.

Restricted cubic spline analyses were conducted to investigate the potential nonlinear dose–response relationships between BHB concentrations and the risk of cognitive decline across global and domain-specific measures. Plasma concentrations of BHB did not show significant dose–response associations with the incidence of global cognitive decline, and a similar result was observed after adjusting for the covariates age, sex, PRS for Alzheimer’s disease, ethnicity, education, income, smoking, alcohol consumption, physical activity, BMI, and comorbidities (Figure 2). According to the cognitive declines in 5 domain-specific measures, the decline in fluid intelligence showed significant and nonlinear associations with plasma BHB concentrations in both unadjusted and adjusted models (unadjusted: *P* for overall = 0.002, *P* for nonlinearity = 0.001, and adjusted: *P* for overall = 0.002, *P* for nonlinearity <0.001). The decline in pairs matching in the unadjusted model showed a significant association with plasma BHB concentrations (*P* for overall = 0.047), although the nonlinearity was not statistically significant (*P* for nonlinearity = 0.066). However, the association was not shown after adjusting for covariates (Supplementary Figure 1).

### 3.3 Changes of cognitive functions by plasma β-hydroxybutyrate

The associations between plasma concentrations of BHB and global cognition score, values of the five cognitive domains, and changes from baseline to follow-up are shown in Supplementary Table 2. The reactive time domain at baseline was negatively associated with plasma levels of BHB (*β* = −0.721, *p* = 0.006); however, after adjusting for the above covariates, the reactive time at baseline was not correlated with BHB levels.

Among the global cognition score and five cognitive function domains, the mean change in fluid intelligence from baseline to follow-up showed a positive increasing trend according to the plasma BHB tertile (*P* trend for unadjusted = 0.028 and *P* trend for adjusted = 0.018) (Table 2).

**Table 2:** Changes of cognitive function by plasma β-hydroxybutyrate tertiles.

Change of cognitive function		Tertile 1	Tertile 2	Tertile 3	*p*	*p* for trend
Unadjusted	△ Pairs matching	0.037 (0.0343)	0.007 (0.0337)	−0.039 (0.0369)	0.301	0.124
	△ Reaction time	0.007 (0.0252)	−0.026 (0.0257)	0.048 (0.0264)	0.124	0.252
	△ Prospective memory	0.062 (0.0330)	−0.006 (0.0323)	−0.021 (0.0324)	0.160	0.073
	△ Fluid intelligence	−0.036 (0.0230)	0.014 (0.0228)	0.035 (0.0223)	0.078	0.028
	△ Numeric memory	−0.018 (0.0275)	−0.006 (0.0280)	0.047 (0.0270)	0.211	0.099
	△ Global cognition score	0.008 (0.0218)	−0.006 (0.0225)	0.031 (0.0222)	0.480	0.451
Adjusted^*^	△P airs matching	0.075 (0.4003)	0.053 (0.4012)	0.008 (0.4013)	0.396	0.182
	△ Reaction time	−0.394 (0.2938)	−0.425 (0.2944)	−0.355 (0.2945)	0.167	0.288
	△ Prospective memory	−0.178 (0.3722)	−0.231 (0.3730)	−0.258 (0.3731)	0.220	0.087
	△ Fluid intelligence	0.071 (0.2598)	0.124 (0.2603)	0.147 (0.2604)	0.054	0.018
	△ Numeric memory	−0.306 (0.3144)	−0.291 (0.3151)	−0.245 (0.3152)	0.265	0.120
	△ Global cognition score	−0.200 (0.2534)	−0.204 (0.2539)	−0.173 (0.2540)	0.567	0.391

△ indicate values for follow-up minus the baseline cognitive function tests.

Values are presented as mean (standard error).

*Adjusted for age, sex, polygenic risk score tertiles for Alzheimer’s disease, ethnicity, education, income, smoking, alcohol consumption, physical activity, body mass index, and comorbidities.

### 3.4 Associations between cognitive decline and plasma β-hydroxybutyrate levels

Over the follow-up period, compared to BHB tertile 1, BHB tertiles 2 and 3 were not associated with the risk of cognitive decline given by the global cognition score. Among the five cognitive domains, there was no increased risk of decline in the values of the pairs matching domain with plasma to BHB tertile 3 compared to plasma BHB tertile 1 after adjusting for covariates (adjusted OR, 1.18; 95% CI, 0.97–1.42), whereas an increased risk for a decline in pairs matching was associated with plasma BHB tertile 3 compared to plasma BHB tertile 1 (unadjusted OR, 1.24; 95% CI, 1.03–1.50). A decreased risk of decline in fluid intelligence was associated with plasma BHB tertile 3 compared to plasma BHB tertile 1 (unadjusted OR, 0.84; 95% CI, 0.73–0.98), and the association remained after adjusting for covariates (adjusted OR, 0.83; 95% CI, 0.72–0.97) (Table 3).

**Table 3:** Associations between cognitive decline and plasma β-hydroxybutyrate levels by tertile.

Outcomes	*n* (%)		Odds ratios (with 95% confidence intervals)			
	Cases	Controls	Unadjusted	*p*	Adjusted^*^	*p*
Cognitive decline	2,143	2,143				
β-hydroxybutyrate tertile 1	724	706	1		1	
β-hydroxybutyrate tertile 2	719	706	0.99 (0.86–1.15)	0.925	0.99 (0.85–1.15)	0.892
β-hydroxybutyrate tertile 3	700	731	0.93 (0.81–1.08)	0.359	0.94 (0.81–1.10)	0.450
Decline, pairs matching	820	3,466				
β-hydroxybutyrate tertile 1	246	1,184	1		1	
β-hydroxybutyrate tertile 2	280	1,145	1.18 (0.97–1.42)	0.092	1.16 (0.95–1.40)	0.142
β-hydroxybutyrate tertile 3	294	1,137	1.24 (1.03–1.50)	0.023	1.18 (0.97–1.42)	0.096
Decline, reaction time	2,129	2,157				
β-hydroxybutyrate tertile 1	704	726	1		1	
β-hydroxybutyrate tertile 2	739	686	1.11 (0.96–1.29)	0.160	1.10 (0.95–1.28)	0.204
β-hydroxybutyrate tertile 3	686	745	0.95 (0.82–1.10)	0.490	0.95 (0.82–1.10)	0.475
Decline, prospective memory	358	3,928				
β-hydroxybutyrate tertile 1	111	1,319	1		1	
β-hydroxybutyrate tertile 2	121	1,304	1.10 (0.84–1.44)	0.476	1.01 (0.77–1.34)	0.916
β-hydroxybutyrate tertile 3	126	1,305	1.15 (0.88–1.50)	0.312	1.07 (0.82–1.41)	0.604
Decline, fluid intelligence	1749	2,537				
β-hydroxybutyrate tertile 1	617	813	1		1	
β-hydroxybutyrate tertile 2	573	852	0.89 (0.76–1.03)	0.112	0.88 (0.76–1.03)	0.110
β-hydroxybutyrate tertile 3	559	872	0.84 (0.73–0.98)	0.027	0.83 (0.72–0.97)	0.018
Decline, numeric memory	1,673	2,613				
β-hydroxybutyrate tertile 1	560	870	1		1	
β-hydroxybutyrate tertile 2	567	858	1.03 (0.88–1.19)	0.730	1.03 (0.89–1.21)	0.660
β-hydroxybutyrate tertile 3	546	885	0.96 (0.82–1.11)	0.582	0.98 (0.84–1.14)	0.753

Cognitive decline was calculated as the global cognitive function score at follow-up minus that at baseline, which indicate a negative value for them.

*Adjusted for age, sex, polygenic risk score tertiles for Alzheimer’s disease, ethnicity, education, income, smoking, alcohol consumption, physical activity, body mass index, and comorbidities.

## 4 Discussion

We matched 2,143 cases and controls in the middle-aged and older age participants in this case–control study nested in the UK Biobank cohort. There was no association between the risk of global cognitive decline and plasma BHB levels, whereas high BHB levels were associated with a slower decline in fluid intelligence.

Previous experimental studies have suggested that BHB plays a role in preventing cognitive decline and Alzheimer’s disease (Myette-Cote et al., 2022; Reger et al., 2004; Roberts et al., 2017), and therapies that elevate circulating BHB, such as a special diet or ketone supplementation, might hold promise for decreasing Alzheimer’s disease and related cognitive decline (McClure et al., 2024a; McClure et al., 2024b; Qin et al., 2023). However, little is known about the impact of basal endogenous BHB status on cognitive improvement or decline (Kim et al., 2025). Therefore, we hypothesized that higher plasma concentrations of BHB within the physiological range observed in the fed, non-fasting state may be associated with preserved cognitive function in middle-aged and older adults without a diagnosis of Alzheimer’s disease or other types of dementia. Our results indicate that circulating BHB status in the body is not statistically associated with global cognitive decline, although higher plasma BHB levels were associated with a slower decline in fluid intelligence. This lack of association could be due to potential residual confounding from unmeasured variables. We analyzed the data after adjusting for covariates that affect the development of cognitive decline, but not enzymes such as β-hydroxybutyrate dehydrogenase and HMG-CoA synthase 2, and the related genetic factors that can influence endogenous BHB status (Hegardt, 1999; Thumelin et al., 1993). Further investigations should clarify the association between endogenous BHB status and cognitive function decline considering these factors as instrumental variables affecting the endogenous BHB status of individuals and lead to proactive intervention to prevent the development of cognitive decline, Alzheimer’s disease, or dementia using personalized prevention strategies. In addition, it is noteworthy that plasma BHB concentrations in the present study were confined to low levels within the physiological range, below those typically achieved in nutritional ketosis or exogenous ketone interventions. Accordingly, our findings should not be interpreted as evidence that increasing BHB concentrations to ketogenic levels was associated with a slower decline in fluid intelligence; rather, they suggest that very low endogenous BHB availability may be associated with greater vulnerability of fluid intelligence in older adults.

In this study, high plasma levels of BHB preserved cognitive function in fluid intelligence, and enhanced the association after adjusting for covariates, including the variable of physical activity (Koeslag et al., 1980), suggesting that the beneficial effect of high endogenous BHB levels on fluid intelligence may be stronger than that on other cognitive function domains. Fluid intelligence is defined as the capacity to solve novel problems and adapt to new situations through abstract reasoning rather than reliance on preexisting knowledge, encompassing higher-order cognitive abilities supported by processing speed and working memory and predominantly mediated by fronto-parietal networks (Kent, 2017). Given its high cognitive and energetic demands, age- or insulin resistance–related impairments in brain glucose utilization may contribute to its decline (Cunnane et al., 2011) BHB readily crosses the blood–brain barrier and can serve as an efficient alternative substrate for ATP production, potentially compensating for reduced glucose availability (Cunnane et al., 2016). Therefore, higher endogenous BHB levels may reflect preserved metabolic flexibility and an enhanced capacity to adapt to cerebral energy deficits, thereby attenuating fluid intelligence decline (Yu et al., 2024). Beyond its role as an alternative energy substrate, BHB functions as a signaling molecule with potential neuroprotective effects; experimental evidence suggests that BHB may inhibit histone deacetylases, upregulate brain-derived neurotrophic factor expression, and reduce oxidative stress and neuroinflammation, thereby supporting synaptic plasticity (Newman and Verdin, 2014; Shimazu et al., 2013). These mechanisms may be particularly relevant to the maintenance of fluid intelligence, which relies on frontal networks involved in working memory and abstract reasoning (Barbey, 2018). In contrast, other cognitive domains assessed, including reaction time and memory-based tasks, such as pairs matching, numeric memory, and prospective memory, may be less dependent on such metabolic flexibility or insufficiently sensitive to detect subtle energy-related effects (Jeong et al., 2017).

This is the first nested case–control study to investigate the associations between endogenous plasma levels of BHB and incident cognitive decline in a large cohort of individuals aged 40 years or older. We used data from the UK Biobank, which allowed us to examine the associations between BHB levels and the risk of cognitive decline after adjusting for possible confounding variables. This study has some limitations. First, the analyses were limited to a single baseline BHB measurement because BHB concentrations were measured only at baseline for the vast majority of participants, although repeated or longitudinal BHB measurements would be informative. Second, we used the standard PRS because of the small number of participants for whom the enhanced PRS was available, despite its better performance of the enhanced PRS (Thompson et al., 2022). Third, potential residual confounding factors were not considered, such as dietary patterns, and the enzymes and single nucleotide polymorphisms of the related genes that can affect endogenous BHB status, and menopausal status that may affect cognitive function.

## 5 Conclusion

The risk of a decline in global cognition from baseline to follow-up was not associated with plasma levels of BHB at baseline, independent of covariates, while a decline in fluid intelligence, one of the five domains used to evaluate global cognitive function, was negatively related to BHB levels by tertiles in middle-aged and older adults. Further studies are warranted to clarify the relationship between endogenous BHB status and cognitive reserve in healthy adults without Alzheimer’s disease or dementia, considering other confounding factors, including a genetic predisposition to endogenous BHB status, or dietary intake or pattern.

## Data Availability

Publicly available datasets were analyzed in this study. This data can be found at: the UK Biobank is an open access resource, and researchers can apply it to the UK Biobank dataset by registering and applying it at https://www.ukbiobank.ac.uk/enable-your-research/register.

## Supplementary material

Supplementary FIGURE 1 Dose-response analysis of plasma β-hydroxybutyrate concentrations and cognitive decline risks in five cognitive function domains. *p* values were determined using restricted cubic spline regression in unadjusted [**(A)** pairs matching, **(B)** reaction time, **(C)** prospective memory, **(D)** fluid intelligence, and **(E)** numeric memory] and adjusted models [**(F)** pairs matching, **(G)** reaction time, **(H)** prospective memory, **(I)** fluid intelligence, and **(J)** numeric memory]. Adjusted for age, sex, polygenic risk score for Alzheimer’s disease, ethnicity, household income, educational level, smoking status, alcohol consumption, physical activity, body mass index, and history of comorbidities.
