# Supplementary figures and images for: Endogenous β-hydroxybutyrate and the risk of cognitive decline: a nested case-control study in the UK Biobank cohort
Source: Front Aging Neurosci. 2026 Feb 11;18:1768532. doi: 10.3389/fnagi.2026.1768532 (PMC12932502; doi:10.3389/fnagi.2026.1768532)

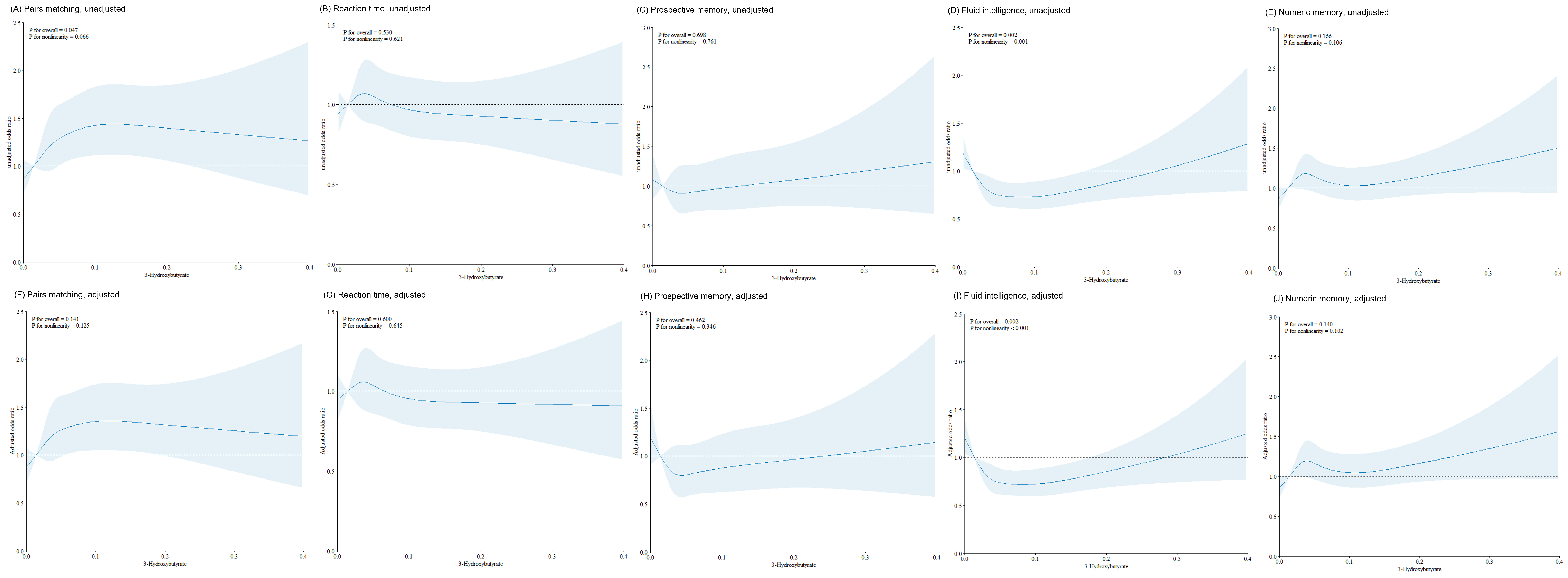

Supplement: Supplementary FIGURE 1 — Dose-response analysis of plasma β-hydroxybutyrate concentrations and cognitive decline risks in five cognitive function domains. p values were determined using restricted cubic spline regression in unadjusted [(A) pairs matching, (B) reaction time, (C) prospective memory, (D) fluid intelligence, and (E) numeric memory] and adjusted models [(F) pairs matching, (G) reaction time, (H) prospective memory, (I) fluid intelligence, and (J) numeric memory]. Adjusted for age, sex, polygenic risk score for Alzheimer’s disease, ethnicity, household income, educational level, smoking status, alcohol consumption, physical activity, body mass index, and history of comorbidities. [file Image_1.tiff]
