# Supplementary material for: Endogenous β-hydroxybutyrate and the risk of cognitive decline: a nested case-control study in the UK Biobank cohort
Source: Front Aging Neurosci. 2026 Feb 11;18:1768532. doi: 10.3389/fnagi.2026.1768532 (PMC12932502; doi:10.3389/fnagi.2026.1768532)
Supplement: Supplementary file 2 [file Data_Sheet_1.docx]

Supplementary Table 1. The scores of global cognition and all cognitive function domains.

| Cognitive function score | Cognitive decline | Controls | *P* |
| --- | --- | --- | --- |
| Global cognition score |  |  |  |
| Baseline | 0.35 (0.85) | -0.35 (1.01) | <0.001 |
| Follow-up | -0.28 (0.99) | 0.31 (0.89) | <0.001 |
| Baseline, raw |  |  |  |
| Pairs matching | 0.16 (0.52) | 0.51 (1.07) | <0.001 |
| Reaction time | 533.7 (97.1) | 553.1 (115.9) | <0.001 |
| Prospective memory | 0.94 (0.23) | 0.83 (0.38) | <0.001 |
| Fluid intelligence | 7.35 (2.04) | 6.53 (1.93) | <0.001 |
| Numeric memory | 7.38 (1.22) | 6.68 (1.38) | <0.001 |
| Baseline, Z-scores |  |  |  |
| Pairs matching | 0.217 (0.628) | -0.218 (1.226) | <0.001 |
| Reaction time | 0.083 (0.936) | -0.097 (1.074) | <0.001 |
| Prospective memory | 0.180 (0.730) | -0.185 (1.188) | <0.001 |
| Fluid intelligence | 0.208 (1.012) | -0.199 (0.957) | <0.001 |
| Numeric memory | 0.263 (0.902) | -0.257 (1.017) | <0.001 |
| Follow-up, raw |  |  |  |
| Pairs matching | 0.50 (1.04) | 0.18 (0.54) | <0.001 |
| Reaction time | 603.9 (110.4) | 567.4 (97.5) | <0.001 |
| Prospective memory | 1.08 (0.42) | 1.02 (0.24) | <0.001 |
| Fluid intelligence | 6.50 (1.99) | 7.16 (2.01) | <0.001 |
| Numeric memory | 6.64 (1.24) | 7.07 (1.22) | <0.001 |
| Follow-up, Z-scores |  |  |  |
| Pairs matching | -0.193 (1.197) | 0.195 (0.684) | <0.001 |
| Reaction time | -0.171 (1.020) | 0.177 (0.934) | <0.001 |
| Prospective memory | -0.183 (1.175) | 0.201 (0.709) | <0.001 |
| Fluid intelligence | -0.153 (0.979) | 0.170 (0.988) | <0.001 |
| Numeric memory | -0.161 (0.988) | 0.181 (0.965) | <0.001 |

The values are presented as mean (standard deviation).

Supplemental Table 2. Association between plasma β-hydroxybutyrate concentration and cognitive decline.

|  | Unadjusted β | *P* | Adjusted β^*^ | *P* |
| --- | --- | --- | --- | --- |
| Pairs matching |  |  |  |  |
| Baseline | -0.078 | 0.762 | 0.042 | 0.871 |
| Follow up | -0.445 | 0.080 | -0.334 | 0.193 |
| △Pairs matching | -0.372 | 0.276 | -0.376 | 0.275 |
| Reaction time |  |  |  |  |
| Baseline | -0.721 | 0.006 | -0.384 | 0.127 |
| Follow up | -0.474 | 0.065 | -0.191 | 0.439 |
| △Reaction time | 0.247 | 0.325 | 0.193 | 0.445 |
| Prospective memory |  |  |  |  |
| Baseline | 0.051 | 0.845 | 0.119 | 0.643 |
| Follow up | -0.007 | 0.978 | 0.061 | 0.808 |
| △Prospective memory | -0.058 | 0.856 | -0.058 | 0.856 |
| Fluid intelligence |  |  |  |  |
| Baseline | -0.367 | 0.157 | -0.206 | 0.401 |
| Follow up | -0.399 | 0.120 | -0.223 | 0.356 |
| △Fluid intelligence | -0.033 | 0.883 | -0.001 | 0.941 |
| Numeric memory |  |  |  |  |
| Baseline | -0.222 | 0.388 | -0.072 | 0.776 |
| Follow up | -0.173 | 0.498 | -0.064 | 0.798 |
| △Numeric memory | 0.048 | 0.857 | 0.008 | 0.976 |
| Global cognition score |  |  |  |  |
| Baseline | -0.462 | 0.074 | -0.179 | 0.462 |
| Follow up | -0.498 | 0.051 | -0.245 | 0.303 |
| △Global cognition score | -0.036 | 0.868 | -0.065 | 0.766 |

△ means values for follow-up minus baseline of cognitive function tests.

^*^Adjusted for age, sex, polygenic risk score tertiles for Alzheimer’s disease, ethnicity, education, income, smoking, alcohol, physical activities, body mass index, and comorbidities
